# Navigating Multiple Sources of Healing in the Context of HIV/AIDS and Wide Availability of Antiretroviral Treatment: A Qualitative Study of Community Participants’ Perceptions and Experiences in Rural South Africa

**DOI:** 10.3389/fpubh.2018.00073

**Published:** 2018-03-12

**Authors:** Thembelihle Zuma, Daniel Wight, Tamsen Rochat, Mosa Moshabela

**Affiliations:** ^1^Africa Health Research Institute, Mtubatuba, South Africa; ^2^School of Nursing and Public Health University of KwaZulu-Natal, Durban, South Africa; ^3^MRC/CSO Social and Public Health Sciences Unit, University of Glasgow, Glasgow, United Kingdom; ^4^Human Sciences Research Council/Human and Social Development (HSD), MRC Developmental Pathways to Health Research Unit, School of Clinical Medicine, University of Witwatersrand, Durban, South Africa; ^5^Discipline of Rural Health, School of Nursing and Public Health, College of Health Sciences, University of KwaZulu-Natal, Durban, South Africa

**Keywords:** HIV/AIDS, antiretroviral treatment, traditional healers, treatment as prevention, health care, South Africa

## Abstract

**Background:**

South Africa introduced the world’s largest antiretroviral treatment (ART) program in 2004 and since 2016 the Department of Health implemented a universal Treatment as Prevention (TasP) strategy. However, some studies have shown that increasing the availability of ART is insufficient for the comprehensive treatment of HIV, since many people still use traditional health practitioners (THPs) to avoid being identified as HIV positive, and for reasons unrelated to HIV/AIDS. This qualitative study explored the factors influencing how both HIV-negative and HIV-positive people choose amongst multiple sources of healing and how they engage with them, in the context of HIV/AIDS and wide availability of ART.

**Methods:**

Data were collected as part of a larger TasP trial at the Africa Health Research Institute, KwaZulu-Natal. Repeat in-depth individual interviews were conducted with 10 participants. Repeat group discussions were conducted with 42 participants. Group discussion data were triangulated using community walks and photo-voice techniques to give more insight into the perceptions of community members. All data were collected over 18 months. Thematic analysis was used to analyze participants’ narratives from both individual interviews and group discussions.

**Findings:**

In the context of HIV/AIDS and wide availability of ART, use of biomedical and traditional healing systems seemed to be common in this locality. People used THPs to meet family expectations, particularly those of authoritative heads of households such as parents or grandparents. Most participants believed that THPs could address specific types of illnesses, especially those understood to be spiritually caused and which could not be addressed or cured by biomedical practitioners. However, it was not easy for participants to separate some spiritually caused illnesses from biological illnesses in the context of HIV/AIDS. These data demonstrate that in this context, the use of THPs continues regardless of the wide availability of ART. To meet the health care needs of those patients requiring a health care system which combines biomedical and traditional approaches, collaboration and integration of biomedical and traditional health care should be considered.

## Introduction

For the past year, South Africa is initiating antiretroviral treatment (ART) with all patients testing HIV positive regardless of CD4 count, in line with the recommendations by the World Health Organization (WHO) (1). The use of ART as prevention, by treating all patients infected with HIV irrespective of their CD4 count has played a significant role in the reduction of AIDS-related deaths globally (2). In South Africa, the world’s largest ART program was introduced in 2004, resulting in significant improvements in mortality and morbidity, and improvements in quality of life (3). Even 30% access to ART and care reduces HIV incidence and significantly reduces adult morbidity and mortality, particularly when ART is initiated early (3–5). It has been demonstrated that with ART, a decline in HIV transmission is effective at both individual and population levels (6, 7). In the launch month of the South African Department of Health’s (DoH) universal Treatment as Prevention (TasP) strategy (8), about 31,157 HIV-positive individuals were initiated, reaching double the standard monthly ART initiations (8).

Alongside biomedical and behavioral interventions aimed at reversing the pandemic, the DoH recognizes the role of traditional health practitioners (THPs) as a pivotal resource in improving HIV/AIDS health programs (9, 10). Traditional healing systems had been dominant prior to the arrival of Western missionaries, and their position remained largely unregulated and free of legislation. However, during the apartheid era, the Medical Association outlawed the traditional healing systems, declaring them illegal and unscientific (11). Within the democratically elected government of South Africa, the South African Traditional Health Practitioners Act acknowledges that the African traditional healing systems provide a holistic healing approach, intertwined with cultural beliefs, which work together to influence wellness, ill-health, and misfortune (12, 13). The process of traditional healing is not only directed as physical care, it focuses on social, psychological, and spiritual aspects of illness (14, 15). As such, the THP becomes part of the socio-cultural life of the members of the community as a whole (16, 17). This notion of illness as both a biological and social phenomenon is illustrated in Germond and Cochrane’s Health-world Framework which reinforces that understanding the experience of illness needs specialized “scientific” healthcare and social and interpersonal meaning (18, 19).

The persistent issues of fear, stigma, and discrimination often prevent people from getting tested, and accessing care, with most (70–80%) seeking healing from the traditional healing system (20–22). Whether THPs are an appropriate resource to support the scaling up of HIV treatment and prevention services in South Africa remains unclear. Even though studies show that THPs provide services to people affected and infected with HIV alongside the biomedical system (23–25), the systems are often presented as being in competition with respect to HIV/AIDS. However, the WHOhas identified a few possibilities for linking biomedical and traditional healing systems. These include incorporation, collaboration, and integration of both systems as part of a primary healthcare approach (13, 26).

Evidence from qualitative and quantitative research suggests that there are challenges for patients who use THPs or both biomedical and traditional healing systems in the context of HIV/AIDS (23, 27–29), and there is limited data on how people navigate multiple sources of health care, including traditional healing systems, in the context of HIV/AIDS and wide scaleup of ART. A multi-country qualitative study found that the use of both traditional and biomedical systems, known as medical pluralism manifests across traditional, faith-based and biomedical health-worlds (30). According to this study, medical pluralism contributes toward delays in HIV-related care and interruption of care for people living with HIV (PLHIV). The study reported that medical pluralism also contributes toward tensions between traditional and biomedical systems driven by fear of drug-to-drug interactions and mistrust between providers (30).

However, some studies have shown that increasing the availability of ART is not enough, as more people still use the traditional healing system to avoid being identified as HIV positive, and for reasons unrelated to HIV/AIDS (25, 31, 32). In South Africa particularly, there is a growing understanding that the broader cultural and social context may influence engagement with biomedical public health care systems or inform the acceptability of lifelong treatments such as ART (23). There is evidence that socio-cultural beliefs and dissatisfaction with biomedical public health systems drives significant levels of medical pluralism and the continued use of the traditional healing system alongside ART care, particularly in rural populations (32, 33). A study conducted in both urban and rural areas in South Africa found that close to 80% of participants utilize THPs before being diagnosed with HIV (34). Other studies found that the majority (67%) of community participants used THPs to facilitate communication between the living (natural) and the dead (supernatural) and for their ability to divine the cause and source of a person’s illness or social problem (35, 36). The term “affliction” is used to refer to the subjective experience of symptoms, which is socially determined, whereas “disease” refers to a biomedical condition (37, 38).

A recent study showed that PLHIV use THPs for needs distinct from the care and treatment of HIV/AIDS (25). Though extensive research has been done to understand the use of THPs in the context of HIV/AIDS, few studies have explored how people navigate multiple sources of healing in the context of HIV/AIDS and wide scaleup of ART (32). Moreover, available literature does not include both HIV-negative and HIV-positive individuals in the same investigation. Available research has been conducted with HIV-positive patients once they have already been diagnosed or when they have started ART (25, 39, 40). In response to this gap in evidence, the current study examines the factors influencing how both HIV-negative and HIV-positive people choose among multiple sources of healing and how they engage with them, in the context of HIV/AIDS and wide availability of ART.

## Methodology

### Study Design and Setting

The study used qualitative methods, involving repeat group discussions (41), repeat semi-structured individual interviews (42, 43), community walks, photo-voice techniques (44), and participant observation (45). Data were collected as part of a larger TasP trial at the Africa Health Research Institute, formerly Africa Centre for Population Health, in northern KwaZulu-Natal, South Africa (46). The TasP trial implemented universal and repeat home-based HIV testing of all resident adults as standard of care and immediate ART initiation was implemented as the intervention. A qualitative study was conducted between January 2013 and July 2014, as part of the TasP trial. The qualitative study explored among various community members, including THPs, access to health care, how HIV/AIDS is managed and approached, as well as local practices that facilitate or hinder HIV testing, ART initiation, and adherence. Group discussions and individual interviews were conducted to explore perceptions of community members over time. Details of the social science sub-studies are provided in Orne-Gliemann et al. (47).

The area in which the study was conducted forms part of the Hlabisa health sub-district, one of the five sub-districts in the district of UMkhanyakude. The area is predominantly rural with one large formal urban township characterized by a high density of households (48). The study was conducted in the rural parts of the area, made up of scattered settlements throughout the district rather than grouped as is the case in other parts of Africa (49). People can access HIV testing, counseling, and ART in public health facilities according to South African DoH guidelines, which indicate that, all HIV-positive children, adolescents, and adults who are willing and ready, should be offered ART regardless of CD4 count (8). HIV prevalence in the Hlabisa sub-district is 24% among adults aged 15–49; and 0.5% among those aged above 50 years (7, 37). Approximately 92% of the population speak *IsiZulu* as a first language (38). One district hospital and 13 fixed primary health care clinics provide primary health care in the Hlabisa sub-district. In addition, there are 30 mobile clinic points that are visited twice monthly and 130 community health workers (CHW), each of whom is expected to regularly visit a group of assigned homesteads (49).

### Study Sample

In each trial cluster, repeat group discussion participants were recruited. The smallest cluster was about 18 km^2^ and the largest about 26 km^2^. Participants recruited from the same cluster were familiar or at least acquainted with each other, and their HIV status was unknown to the facilitator (TZ). Groups were organized as follows: (1) 15 mixed sex younger adults aged between 18 and 35 identified by randomly approaching households across the trial cluster; (2) 16 mixed sex older adults aged >30 recruited across the trial cluster with the help of a CHW who worked in the community to assist with health care and promotion at a household level as part of primary health care delivered by the DoH (49); and (3) 11 mixed sex 18–65-year-olds which included two THPs recruited with the help of a community member who worked at a local creche who knew the THPs and referred the facilitator to them, she was not part of the group discussion.

Repeat individual interviews were conducted with six women and four men aged over 16 years living in the TasP trial clusters. Five HIV-positive individuals were purposefully recruited from trial clinics, these individuals had been diagnosed through home-based HIV testing before being referred to TasP clinics as part of the TasP trial (50), two in intervention arms of the trial and three in control arms of the trial. Five respondents were recruited by randomly approaching households across the trial clusters, of whom three were HIV positive, one HIV negative, and one had never tested for HIV, thus having an unknown HIV status.

### Data Collection

**Figure 1:**
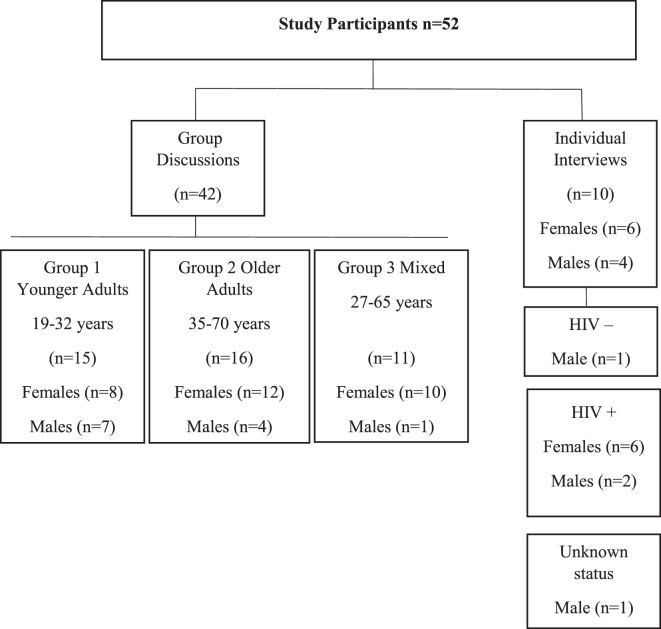
Participants’ profile.

All interviews and group discussions, illustrated in Figure 1, were conducted by the first author, a PhD candidate who speaks *IsiZulu* as her first language and has worked and resided in the study area for 8 years. She explained the purpose of the study during recruitment to each participant. Interviews and group discussions explored broad topics which were not mutually exclusive, summarized in Table 1. Subsequently, related probes were incorporated to complement the narrative of each participant or group. Four group discussions were conducted with each group lasting 60–120 min. Venues for group discussions were prearranged through local *izinduna* (traditional headsmen); they included a community hall, a school hall, and a building connected to a local informal food trader. Three repeat interviews were conducted either at the participants’ home or TasP clinic, depending on the participants’ choice and lasting for 30–60 min.

**Table 1:** Overview of sample and topics covered over repeat individual interviews and repeat group discussions.

Individual interviews	IDI 1	IDI 2	IDI 3		
Attendance over repeat in-depth interviews	*n* = 10 (M4/F6)	*n* = 10 (M4/F6)	*n* = 9 (M4/F5)		

Topic: Content	Access to health care in the community and knowledge of HIV status	Stigma induced by attending Treatment as Prevention (TasP) trial clinics. Social support and disclosure	Understanding of benefits of TasP, facilitators and barriers of testing and ART		

Approach used	Personal and shared experiences: in-depth semi-structured interviews	Personal and shared experiences: in-depth semi-structured interviews	Personal and shared experiences: in-depth semi-structured interviews		

**Focus group discussions**	**FGD 1**	**FGD 2**	**FGD 3**	**Community Walk**	**FGD 4**

Attendance over repeat group Discussions	Younger adults *n* = 15 Older adults *n* = 16 Mixed group *n* = 11	Younger adults *n* = 12 Older adults *n* = 11 Mixed group *n* = 10	Younger adults *n* = 11 Older adults *n* = 12 Mixed group *n* = 9	Younger adults *n* = 6 Older adults *n* = 12 Mixed group *n* = 9	Younger adults *n* = 10 Older adults *n* = 10 Mixed group *n* = 10

Topic: Content	Health care services in the community	Community and individual experiences and perceptions of TasP	Local cultures that facilitate and support regular and repeat testing and HIV status disclosure	Walking around the community with participants to places where they took photos of things they perceived as barriers and facilitators for testing and treatment	Formal discussions about facilitators and barriers to HIV testing and ART uptake

Dates	22/02/2013	30/05/2013	31/07/2013	09/10/2013	13/11/2013

Approach used	Group narratives	Group narratives	Group narratives	Community walk, informal discussions, observation, and taking of photos	Panel discussion of community walk and photos taken by each group discussion participant

Fewer men were recruited as they were either absent from their homes during recruitment, or unable to commit to participating. One interviewee withdrew from the study when she found employment. Twelve individuals withdrew from group discussions when they relocated or found employment away from the trial communities. As a token of appreciation for participants’ time and transport reimbursement for those who traveled to participate, ZAR 50 was given to each participant (after each interview/group discussion). A small lunch pack (juice, fruit, and sandwich) was also provided for group discussion participants (51, 52).

Data were triangulated using community walks and photo-voice techniques to give more insight into the perceptions of community members and to complement their narratives as well as to recognize inconsistencies in the data. The community walk was undertaken with group discussion participants to further understand community characteristics and activities. Photovoice, a visual participatory method was used to facilitate a deeper and nuanced understanding of research phenomena from the participant’s view and to engage and enable participants to communicate creatively through a deeper and more reflexive process, as well as to capture social intersections that shape the experience of health care (53, 54).

Prior to the community walk, at the end of the third discussion for each group, the facilitator discussed the photovoice technique and process with the participants. The discussion included information around how participants were going to capture images, sharing a digital camera, of what they considered a barrier or facilitator to HIV testing, treatment, adherence, and retention to care. During the community walk, the facilitator had minimum interference with the process, only asking questions for clarity to capture field notes. Images only served as raw data for qualitative analysis and used in group discussion four to lead discussions. Images captured by participants during the community walk included but were not limited to, images of available health care services in the community, images illustrating participants’ knowledge and beliefs such as images of traditional medicines and images illustrating traditional rituals. At the beginning of the fourth group discussion, all the participants who had participated in the photo-voice were handed their images, printed and laminated in A4 size paper. One participant’s images in Group 1 (participant 14) were not saved on the camera, and thus not printed for group discussion four, but he was able to discuss the photos he had captured. Most of the participants captured the same images more than once and, in the discussion, only one of each image was used.

### Data Analysis and Interpretation

**Figure 2:**
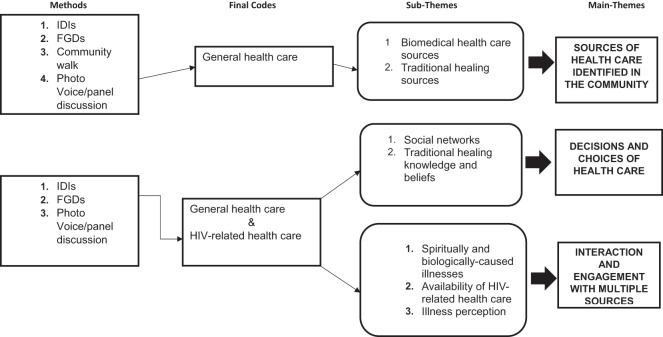
Multiple sources of healing in the context of HIV/AIDS and wide availability of antiretroviral treatment.

The study used thematic analysis to analyze participants’ narratives from both individual interviews and group discussions (55). The voice-recorded interviews and group discussions were translated from *IsiZulu* to English by a translating panel and the first author (Thembelihle Zuma) conducted quality checks of all transcripts, ensuring consistency in language use. Transcripts were read several times by Thembelihle Zuma, to determine common elements, patterns, and themes within interviews and group discussions. Transcribed data were linked to images produced during the community walk in order to add depth to transcriptions and in data analysis. A schematic figure (Figure 2) indicates how analysis was carried out to code across and between different sources of data in order to gain an overview of some of the key issues related to general health care access and HIV-related health care. Transcripts were independently reviewed by Thembelihle Zuma and discussed with one of the co-authors (Mosa Moshabela) to confirm the identified codes. Once saturation was reached, codes were collated into potential themes. Relevant data were extracted from transcripts and summarized in charts according to the identified themes manually in an Excel spreadsheet. Initial themes were then revised in line with the data. Once a satisfactory map of data was achieved, themes were further defined and refined to identify sub-themes. Salient themes were further delineated during write-up to ensure that consensus was reached by the four authors. Differences were resolved through discussions until consensus was reached.

Direct quotes from participants are used to illustrate the findings. No real names of participants were used in this study to maximize confidentiality. Each participant was allocated a pseudonym. Within quotations, text which is placed inside brackets () translates Zulu words, while text inside square brackets [] represent the facilitator’s (a Zulu speaker) descriptions.

### Ethical Considerations

This study was carried out in accordance with the recommendations of the University of KwaZulu-Natal Biomedical Research Ethics Committee (BREC) in 2011 (REF: BFC 104/11) with written informed consent from all subjects. All subjects gave written informed consent in accordance with the Declaration of Helsinki. The protocol was approved by BREC. A further approval was sought for the full protocol developed for the social science sub-studies, separate to the approval granted for the Trial in 2012 (REF: BE090/12). An additional full protocol for the PhD study, separate from the social science sub-studies, was developed and ethical clearance was granted in 2015 (REF: BE432/15). All the participants were asked for consent to participate in the study and to audio-record group discussions and individual interviews.

## Results

### Community Participant Demographics

A total of 52 participants were included in this study (Figure 1). One participant among in-depth interviewees was employed full-time, four among participants in group discussions were employed part-time, and all other participants in both interviews and group discussions were unemployed. None of the participants had received any tertiary level education, 15 had received secondary level education, 17 had received primary level education, and 19 had not received any formal education. Three main themes emerged from the data: (a) sources of health care identified in the community; (b) decisions about choice of health care; and (c) interaction and engagement with multiple sources.

#### Sources of Health Care Identified in the Community

In both individual interviews and group discussions, participants reported that there were different sources of healing, including sources from biomedical and traditional healing systems. Healing sources were also demonstrated through images captured during community walks.

### Biomedical Health Care Sources

Among biomedical health care sources, participants identified two primary health care clinics (PHC), located in two of the trial clusters, and four TasP trial clinics located one in each of the trial clusters. To access PHC, 60.8% of the population walk to PHC facilities, 38.8% use public transport, and 0.4% use their own transport (56). Walking time to the nearest clinic is 81 min and 65% of homesteads travel >1 h to attend PHC (56). Furthermore, health care from private doctors and self-medication obtained from local pharmacies was reported, though not located within participants’ communities.

Nomzamo IDI 1: ehhh…. [silent] this is a very difficult area, others go up the hill to *Ntondweni* [local area name] clinic and others go to *Madwaleni* [local area name] clinic. I used to collect my (HIV) treatment from *Ntondweni* clinic before the arrival of *ukuphila kwami ukuphila kwethu* (my health for our health, TasP clinic) then I decided to come here, it is better to go to *KwaShunqa* [local area name] instead of going up the hill to *Ntondweni* clinic. Right now, I use the clinic in *kwa shunqa* [TasP clinic] (Female, 35 years, unemployed, HIV positive, recruited at a TasP clinic, diagnosed in 2008).

Even though most participants reported that biomedical health care sources were hard to reach, as indicated in the narrative above, other participants said that the availability of HIV-related health care from TasP trial clinics significantly improved access, and subsequently utilization of biomedical treatment and care for PLHIV. In the trial communities, participants were offered home-based HIV testing which improved HIV testing uptake (57). TasP trial clinics were situated at the center of each trial community as the trial considered minimizing distance between participants homes and the clinic and improving clinic attendance by only catering for PLHIV (57). Some participants recognized the local PHC as the only health care source that could respond to community member’s health care needs, rejecting other forms of health sources that were described by others. One of the participants who did not think that other sources of health care were beneficial said:

Thandiwe FGD 1: Our clinic is *Ntondweni* [local area name] clinic. The clinic is very helpful when we are very sick even if someone is being carried by a car or a wheelbarrow, she will visit the clinic and become healthy again. We are able to get any treatment we need, and we get life from that clinic. The clinic is not just helping us, we think it is the only place where we can get assistance because of the way it is helping us, the *Ntondweni* clinic. I do not know anything about traditional herbs, they are no longer used. Now only the new (biomedical) medicine works (female, unemployed, 65 years old, HIV status unknown, recruited from the community).

Two respondents in individual interviews said:

Lindiwe IDI 2: No, I only use medication from the clinic (female, age 46 years, recruited from a TasP clinic, HIV positive, tested in 2012).

Nomzamo IDI 1: at the clinic, we were told (by nurses) not to use (HIV) treatment with any other things (traditional healing/medicines) (Female, 35 years, HIV positive, recruited at the clinic, first tested in 2008).

The use of biomedical health care sources was most commonly reported among respondents living with HIV in individual interviews than it was reported by HIV-negative respondents and participants in group discussions whose HIV status was unknown. Out of eight respondents living with HIV, one female respondent had accessed health care from a THP before she learnt about her HIV status, and one male respondent continued to use both biomedical and traditional health systems concurrently, regardless of his HIV status. Almost all the respondents living with HIV in this study had been on ART for more than 5 years, only one respondent was diagnosed and initiated ART in the TasP trial, thus on ART for less than 5 years.

Common ailments reported by participants as those which could only be diagnosed and treated through biomedical health care included HIV testing, HIV treatment and care, diagnosis, treatment and management of diabetes, blood pressure, tuberculosis (TB), arthritis, and stroke.

Patricia FGD 1: They (traditional medicines and/or THPs) are not able to treat this disease (HIV). This disease (HIV) can only be controlled by tablets. It can only be controlled by tablets not traditional medicines (female, unemployed, 53 years, HIV status unknown, recruited from the community).

Participants also reported ailments which they perceived as minor, only diagnosed and/or treated in biomedical health care sources which included treatment of the eyes. Out of 27 community members who participated in the community walks, 19 captured one or more images which included PHC facilities, TasP trial clinics and containers of medicines found lying around the community, perceived to have been bought from pharmacies as part of self-medication.

### Traditional Healing Sources

Participants identified three types of THPs: diviners, herbalists, and spiritualists. Diviners predominantly use water, ash, salt, and candles to intervene and communicate with the spiritual world on behalf of their patient. Herbalists predominantly use indigenous plants, bones and stones to provide remedies for individuals whom they may have diagnosed themselves, or those that are referred for specialized care by other herbalists or diviners. Spiritualists use Godly spirits and ancestors to learn about people’s problems (58). Among those who used THPs, there was a strong conviction that illnesses could be cured by only visiting a THP. There was also acknowledgment that they did not know modern medicine, thus indicating that they were more comfortable using what they knew and believed in.

Nonhlanhla FGD 1: We use traditional healers because we do not know anything about these modern medicines. A person used to be cured from illness by only going to a traditional healer (female, age above 65, HIV status unknown, recruited from the community).

Some participants said that in their families, they only used THPs. Others added that traditional healing sources were easily accessible, thus utilized frequently.

Sbusiso INT 2: but I don’t go to the clinic because in my family we use traditional medicine (male, 17 years old, recruited from the community, HIV negative, tested from TasP).

Participants further explained that they had knowledge of different traditional herbs which they used as part self-treatment for different ailments.

Khanyisile FGD 1: We are able to use traditional herbs (medicines) available from our community if we have not yet visited the clinic for blood tests. These are the traditional herbs that we listed that people usually use (Female, 21 years old, unemployed, HIV status unknown, recruited from the community).

Among group discussion participants, it was reported that men used the traditional healing system more than women in the community. This was explored in group discussions with both younger and older adults.

Mcebo FGD 1: Men use traditional medicine more (male, 57 years old, HIV status unknown, recruited from the community).

Nhlanhla FGD 2: as men we use traditional medicine, we use *izinsizi* (ashes) and we use an enema [with traditional medicine] to stay healthy (male, 22 years old, HIV status unknown, recruited from the community).

In both individual interviews and focus group discussions, participants said that THPs were unable to cure HIV/AIDS and should not be used by those who were seeking a cure. Participants acknowledged that there were individuals posing as THPs who reported being able to cure HIV/AIDS and PLHIV were known to have died from being deceived by those reporting to have a cure.

Nomathamsanqa FGD 3: What may be missing is that there are still people who are lying, and they are telling the community that they are able to cure HIV. We have never seen anyone up until this day who has been cured of this illness from *imbiza* (mixed concoction of traditional medicines) that they got from a healer to drink or to administer with an enema and got well. I have not heard any of that. We know a lot of people who have died. We have a lot of traditional healers among us. That information is not true, they cannot cure HIV (female, 65 years old, HIV status unknown, recruited from the community).

Participants reported that there were ailments which could only be diagnosed and treated within the traditional healing system. These included illnesses caused by ancestral wrath, which manifested as physical illnesses. Often individuals suffering from such illnesses first sought health care from biomedical health facilities.

Sibusiso INT 3: they go to them (biomedical care) for all kinds of illnesses because you may find that a person goes to the clinic but they do not see the difference, or maybe they go to a hospital and don’t get help so when they come back they try a traditional healer and use traditional herbs (male, 17 years old, recruited from the community, HIV negative, tested from TasP).

Nombuso FGD 3: There is a belt (shingles) that you get due to some spirits (ancestral). Sometime ancestors want something from you and then they expose you to a disease (female, 24 years old, HIV status unknown, recruited from community).

Other ailments which most community members used traditional medicines for, included minor skin problems.

Ndabezinhle IDI 1: We use traditional herbs to bath when we have skin problems. We don’t usually use the clinic for that (male, 64 years old, never tested for HIV, recruited from the community).

Among HIV-positive respondents in individual interviews, only one used both biomedical and traditional healing systems concurrently. The participant who used both systems had disclosed during an interview that he had an ancestral calling and, thus, needed to consult with THPs, however he continued to take ART and his calling did not interfere with ART. There was acknowledgment among participants that because of their heterogeneity, community members used different “things.”

Nomzamo IDI 2: you see people are different and they use different things. Some may go to the clinic others may prefer going to a *Sangoma* (diviner) (Female, 35 years, HIV positive, recruited at the clinic, first tested in 2008).

#### Decisions and Choices of Health Care

Health care decisions and choices were considerably influenced by four main sources: social networks, traditional healing knowledge and beliefs, illness perceptions, and illness experiences.

### Social Networks

Information regarding decisions and individual choices of health care were influenced by different sources, including messages conveyed through participants’ networks, particularly elders in families. Participants perceived members of their community-based networks, including friends and partners, to have some expertise in different forms of illnesses and their treatment. Among both interviewees and group discussion participants, traditional healing was largely endorsed by the elders who often held authority as heads of the households and senior family members. These elders were likely to impart knowledge about traditional healing to their children and other younger family members. In one group discussion with young adults, a female participant presented a dilemma faced as young people growing up in the era of HIV and ART.

Nhlakanipho FGD 2: It [HIV testing] is a last option for our parents because they grew up without the HIV epidemic. They only know that a sick person must wear a goat-skin wristlet or visit a traditional healer. Testing for HIV is their last option since they did not experience it in olden days. This HIV came in our time. (Youth, FGD 2, male, unknown HIV status, 24 years old).

### Traditional Healing Knowledge and Beliefs

Healthcare-seeking decisions are based on past experiences and local knowledge of traditional healing perceived to be effective. Even though it was older adults who were mainly responsible for making healthcare decisions, divergent knowledge, and beliefs about illnesses occurred between family members, which often led to difficulty in determining what was best for the sick family member. The conflict of views occurred with respect to beliefs about the cause and source of illness. Since HIV was described by participants as a “new” and complex illness, there were tendencies toward debate and disagreement about the best treatment and care options. Using an image of a goat (representing traditional rituals) taken during the community walk, a female participant captured this conflict.

Thembani FGD 4: This is a photo of a goat. We, black people, when you lose weight you may be infected with HIV and need to go to the clinic, but your family or parents will instruct you to take the goat and visit a traditional healer. I can say this picture of a goat represents rituals which are not good for people who are HIV positive but have not tested (Mixed, FGD 4, female, unknown HIV status, 62 years old).

Group discussants resolved that, with regard to HIV/AIDS, conflicts in healthcare decision-making among family members could be dealt with by first ascertaining the HIV status of the sick person before THPs are consulted.

Rose FGD 2: If you are possessed with ancestral spirits, you must first go and test for HIV and take treatment if you test positive (Youth, FGD 2, female, unknown HIV status, 25 years old).

### Illness Perception

The perception of illness as assessed by the patient or family members determined the type of healthcare provider to be sought. Choices and decisions of healthcare were often based on distinctions made between illnesses emerging from within the body (biologically caused) and outside the body (spiritually caused). Families and individuals who were disposed to traditional healing sources tended to use traditional explanations of illness and this had implications for the type of health care sought. For example, a young female participant in one group discussion shared this experience:

Khetho FGD 3: I used to have shortness of breath. When I am about to sleep, I would feel like I am suffocating, and I wouldn’t be able to sleep. I consulted traditional healers and they told me that I have *imimoya* (ancestral spirits), and I needed to do *amagobongo* (a ritual to appease ancestors). I did not agree to do *amagobongo* until I met a different traditional healer who gave me this rope that I wear [around the waist], and since then I have never experienced any of that (Youth, FGD 2, female, unknown HIV status, 30 years old).

Participants’ perceptions of illness were complex. In instances where there was no clear distinction between spiritually and biomedically caused illnesses, either THPs or biomedical providers were consulted, and ultimately, both types of systems could be used.

Nomzamo INT 1: it was *umthandazi* (spiritualist), because he [my husband] was sick with several illnesses, because at first it was hiccups and he came back home [after consulting a spiritualist]. At first, he was attended by a traditional healer, *umthandazi*, using only water then he was healed. Then he had *izibhobo* (sharp pains around the abdomen). He stopped using sanctified water when the hiccups stopped. Once he was told (diagnosed) about HIV, he wasn’t continuing with *umthandazi*, he was taking TB treatment at 7am and at 8 pm he took his ARVs (interview 1, female, HIV positive, 35 years old).

## Interaction and Engagement with Multiple Sources

### Spiritually and Biologically Caused Illnesses

HIV was understood to manifests in different ways, including what participants described as “*izifo zonke*,” a combination of illnesses that occurred one after another or at the same time. As a result, the confusion between HIV-related illnesses and spiritual illnesses were often resolved by using both biomedical and traditional healing systems, particularly when it was unclear whether those who were sick suffered from illnesses that only required treatment from a biomedical or traditional healing system.

Nombuso FGD 2: This thing is the same, when you have *imimoya* (ancestral spirits) you lose weight and when you are infected with HIV you also lose weight. When you have *imimoya* you can suffer from sore feet and when you are infected with HIV you can suffer from sore feet. You can have *imimoya* and be infected as well, and suffer from these illnesses, even cracked feet (Youth, FGD 2, female, unknown HIV status, 24 years old).

Participants mentioned that there were diseases which were sent by ancestors, though they manifested as physical illness, their source was spiritual. One participant said:

Nombuso FGD 3: There is a belt (shingles) that you get due to some spirits (ancestral). The one which can be treated by incisions and then it is cured P8: Sometime ancestors want something from you and then they (ancestors) expose you to disease. (FGD 2).

However, in the same group discussion, other participants added that HIV could not be sent or transmitted by ancestors or spiritual powers. Although it was not always clear what participants thought in the group discussions, the predominant view was that HIV was a physical illness not caused by ancestors.

Khetho FGD 2: I think ancestors are not able to infect us with HIV since HIV is a transmitted disease and is transmitted in a certain way. So, it cannot be transmitted by ancestors because we get it in a different way (FGD 2).

### Availability of HIV-Relevant Health Care

Benefits of ART were fully acknowledged by participants in both interviews and group discussions. Both HIV-positive and negative-participants gave examples of how ART had changed the lives of many people and decreased death rates. They pointed out that when one was using traditional and biomedical health systems concurrently or alternatively, it was important to consult with THPs who were registered and trained in HIV/AIDS. Analysis of data from THPs in the same setting found that THPs who had not received formal HIV training were considered HIV incompetent (59). It was easy to identify trained THPs as they had certificates hung on the walls of their consultation rooms, and most of them performed safer practices due to years of encounters with HIV-positive patients. Participants’ concerns around using both systems concurrently in the context of HIV/AIDS were related to drug interactions, drug absorbency, and THPs’ ability to take precautions so that HIV would not be transmitted between patients.

Thembani FGD 1: The registered traditional healer will not tell you to stop taking your treatment when you visit him. If you take your treatment at 8h00 then you can take your traditional medicine around 11h00-12h00 (Older, FGD 1, female, unknown HIV status, 32 years).

Some participants, particularly in individual interviews, considered HIV/AIDS as one of the “new” illnesses which could not be handled within the traditional healing system and they held THPs responsible for misdiagnosing those infected with HIV and for prescribing treatment that caused harm instead of helping PLHIV. An individual interview participant said:

Thabile INT 1: No, I think it [traditional healing] is bad. They [THPs] sometimes refer a person to initiation [training process to become a traditional healer] and use *amagobongo* (type of traditional medicinal herbs mixed together) which destroys people who have HIV. They [THPs] instruct the person to regurgitate and will end up dying. People will say it is ‘*idliso*’ (poisoning) and go to a healer to be cured from *idliso*, but it is just HIV (individual interview 1, female, PLHIV, 35 years old).

In group discussions, participants held different views. Some emphasized the significance of THPs as custodians of cultural beliefs and rituals. They said that health care from THPs could be sought regardless of HIV status.

Thabo FGD 3: we will never stop doing these things [traditional rituals and practices]. If you don’t do it where you live, here we do these things because they work (younger, FGD 3, male, 20 years old).

Other participants did not entirely disapprove of traditional medicines, rituals, or practices. By not doing so, they did not report that they endorsed the system. Their view was that both traditional healing and biomedical health care were significant and necessary to address different health care needs for the benefit of the patient. Those participants who were comfortable integrating both health care systems stated that THPs had adopted ways of handling “new” illnesses, had acquired training, registered with the traditional healers’ organization, and thus were credible. One participant stated:

Mphikeleli FGD 4: Some traditional healers have certificates and some don’t have but most of them know that they cannot use one razor blade on multiple patients because of HIV. They destroy the razor blade after using it on each person. Sometimes they divide one blade into small pieces and use each piece on one person only and then they throw it away (Older, FGD 4, male, 70 years old).

Participants who used both health care systems acknowledged the different approaches offered by practitioners, applied practical measures to navigate both systems, and used treatments offered in both concurrently or alternatively. Participants who integrated also reported that some traditional and biomedical medicines could be used together and not cause complications or harm, particularly for PLHIV.

Edna FGD 4: *Nsukumbili* [name of traditional medicine] is not harmful and you can use it with your HIV treatment (Mixed, FGD 4, female, 62 years old).

## Discussion

This qualitative study adds to the literature about factors driving the use of both biomedical and traditional healing systems in the context of HIV/AIDS and wide-scale rollout of ART (25, 32, 60). Previous studies in South Africa have demonstrated that engagement with THPs in the context of HIV/AIDS drives delays in HIV diagnosis and treatment initiation and predicts lowered ART use and retention in care (61–63). In this study, we found that community participants utilized THPs to meet expectations of their social network, particularly expectations of family members who held authority as heads of households such as parents or grandparents. A study conducted in the Limpopo province of South Africa demonstrated that parents and grandparents are key sources of traditional healing knowledge in their families (64). Most participants believed that THPs could address specific types of illnesses, particularly those understood as spiritually caused and could not be addressed or cured by biomedical practitioners. Participants held this view regardless of knowledge of HIV status. Friends and partners who had expertise or experience with the traditional healing systems were also influential in facilitating use of THPs. Moreover, participants fully acknowledged the value of ART. Some reported that THPs were not competent to treat HIV/AIDS, but others integrated both biomedical and traditional healing methods to handle illness in the context of HIV.

While some participants focused on the physical aspects of health, others focused on aspects of health covering physical, social, and spiritual well-being. Relations with family, other members of participants’ social networks and partners were considered important and an essential indicator to the utilization of THPs. Germond and Cochrane consider six overlapping concepts of holistic health and well-being: the person, the family, the village, the nation, religion, and the earth (65). Health-world is used to explain the complexity of health beliefs and behaviors, which include both social and religious dimensions (54), and explains interactions between biomedical and traditional healing systems. While biomedicine is primarily concerned with recognizing and treating disease, the traditional healing system seeks to provide meaningful explanations for illness and respond to the personal, family, and community issues surrounding illness (65).

This study draws on the work by Germond and Cochrane which distinguishes between physical health and wider aspects of health that include social and religious dimensions. Even when talking about HIV/AIDS, participants considered aspects of health that went beyond the physical well-being and talked about their cultural rituals and cultural beliefs. THPs were not used as an alternative to ART, rather, they were used to respond to different health care needs (25, 61). In this study, participants’ strong beliefs about illness causation drove seeking care from both systems, concurrently or alternatively. THPs were often used to treat supposed spiritually explained afflictions and biomedical practitioners were used to treat physiologically caused illnesses.

We also found that it was not easy for community participants to separate some spiritually explained afflictions from physiologically caused illnesses in the context of HIV/AIDS. Unfortunately, our data do not allow us to clarify whether participants thought that, in some circumstances, spiritual powers could lead to HIV infection: this remains to be explored. Participants explained that suffering from several illnesses at the same time could be caused by both HIV and spiritual entities. Symptoms, such as weight loss, sore, and cracked feet, were linked to illnesses caused by ancestral spirits (spiritual) as well as HIV (physiological). This lack of clarity led to alternative/concurrent utilization of biomedical and traditional healing systems. In the context of HIV/AIDS and wide availability of ART, utilization of biomedical and traditional healing systems seemed to be common in this community. For example, participants reported that THPs trained in HIV/AIDS offered traditional healing services that did not put those who contacted THPs at risk of contracting HIV/AIDS. Available data on HIV/AIDS-trained THPs demonstrate their ability to provide community-based HIV care, promoting and distributing condoms, and coordinating ART adherence support groups (59, 66, 67). However, other studies have shown that utilization of both biomedical and traditional healing systems among PLHIV predicts non-ART use (28, 68). Some research studies have demonstrated that biomedical health practitioners have negative attitudes toward the utilization of THPs among PLHIV (62, 69).

In this study, participants were concerned about drug interactions and drug absorbency for PLHIV who used both biomedical and traditional healing systems. This study shows that we cannot easily separate biomedical and traditional healing systems in the context of HIV/AIDS. Participants in this study reported that these two systems were used for distinct health care needs. However, in the context of HIV/AIDS, participants reported concerns about similarities between some HIV-related and spiritually caused illnesses, as well as drug interactions and absorbency for PLHIV who utilize both systems. A structure that outlines how traditional and biomedical health care systems can function together (70), while recognizing their independent existence (biomedical vs spiritual aspects of illness), could facilitate sustainable ART rollout. A few possibilities for linking biomedical and traditional healing systems have been identified, involving incorporation of THPs into primary health care, which was suggested by the WHO as part of a primary health care approach (13, 26); collaboration and integration of both biomedical and traditional health care where patients would commonly not receive either a pure biomedical or traditional treatment, but a combination of the two (71–76).

### Limitations

All the data are based on participants’ accounts, and thus affected by how participants chose to represent themselves. However, images in group discussions were used to try and illuminate inconsistencies within data. Participants resided in the same community and, therefore, knew each other: this could potentially have led to social desirability bias. There was a low contact rate for men in this study with only a few participating. Nevertheless, we repeated both interviews and group discussions over time with the same participants and triangulated data sources to enhance rigor and to gage whether views changed over time. The quality of participation in photo-voice varied among focus group participants, 12 participants did not take part in the photovoice and their experiences were, thus, not captured in images. However, participants did not miss more than two meetings out of the five that took place. We acknowledge that data were not collected on some important factors likely to shape the navigation of health care, such as the cost of consulting different practitioners and the extent to which other community members shaped choices in health care.

## Conclusion

This study demonstrates that THPs are an important health care resource in rural South Africa. It is, thus, important to identify possible specific roles of biomedical practitioners and THPs in HIV/AIDS care and treatment. In addition, it is important to identify overlapping roles between the two health systems so that we can improve the quality of information around illnesses or conditions related to HIV that are treated within both systems. The study indicates that family members’ influence regarding health care decisions plays an important role in the type of health care sought by patients. There is, therefore, a need to recognize the importance of socio-cultural and spiritual beliefs of patient’s wider networks. Issues raised in this study suggest a delay in successful wide-scale implementation of ART at a population level as issues related to concurrent and/or alternative utilization of both biomedical and traditional healing systems remain unresolved. This demonstrates that the WHO’s strategy to integrate biomedical and traditional healing systems could provide a culture-sensitive response for users of both biomedical and traditional healing systems who are living with HIV. This study was unable to answer other important factors likely to shape the navigation of health care, and this has been acknowledged in the limitations.

## Ethics Statement

This study was carried out in accordance with the recommendations of the University of KwaZulu-Natal Biomedical Research Ethics Committee (BREC) in 2011 (REF: BFC 104/11) with written informed consent from all subjects. All subjects gave written informed consent in accordance with the Declaration of Helsinki. The protocol was approved by BREC. A further approval was sought for the full protocol developed for the social science sub-studies, separate to the approval granted for the Trial in 2012 (REF: BE090/12). An additional full protocol for the PhD study, separate from the social science sub-studies, was developed and ethical clearance was granted in 2015 (REF: BE432/15). All the participants were asked for consent to participate in the study and to audio-record group discussions and individual interviews.

## Author Contributions

TZ designed the study, collected and analyzed the data, and wrote the first draft. DW contributed to data analysis, supervised the PhD study, and revised the manuscript. TR contributed to the study design and revised the manuscript. MM contributed to data analysis, oversaw writing of the first draft, supervised the PhD study, and revised the manuscript. All authors read and approved the final manuscript.

## Conflict of Interest Statement

The authors declare that this research was conducted in the absence of any commercial or financial relationships that could be construed as a potential conflict of interest.
